# Multidisciplinary approach to assess the toxicities of arsenic and barium in drinking water

**DOI:** 10.1186/s12199-020-00855-8

**Published:** 2020-05-27

**Authors:** Masashi Kato, Nobutaka Ohgami, Shoko Ohnuma, Kazunori Hashimoto, Akira Tazaki, Huadong Xu, Lisa Kondo-Ida, Tian Yuan, Tomoyuki Tsuchiyama, Tingchao He, Fitri Kurniasari, Yishuo Gu, Wei Chen, Yuqi Deng, Kanako Komuro, Keming Tong, Ichiro Yajima

**Affiliations:** 1 Departments of Occupational and Environmental Health, Nagoya University Graduate School of Medicine, 65 Tsurumai-cho, Showa-ku, Nagoya, Aichi 466-8550 Japan; 2 Voluntary Body for International Health Care in Universities, 65 Tsurumai-cho, Showa-ku, Nagoya, Aichi 466-8550 Japan

**Keywords:** Arsenic, Barium, Comprehensive study, Drinking water, Toxic elements, Remediation

## Abstract

Well water could be a stable source of drinking water. Recently, the use of well water as drinking water has been encouraged in developing countries. However, many kinds of disorders caused by toxic elements in well drinking water have been reported. It is our urgent task to resolve the global issue of element-originating diseases. In this review article, our multidisciplinary approaches focusing on oncogenic toxicities and disturbances of sensory organs (skin and ear) induced by arsenic and barium are introduced. First, our environmental monitoring in developing countries in Asia showed elevated concentrations of arsenic and barium in well drinking water. Then our experimental studies in mice and our epidemiological studies in humans showed arsenic-mediated increased risks of hyperpigmented skin and hearing loss with partial elucidation of their mechanisms. Our experimental studies using cultured cells with focus on the expression and activity levels of intracellular signal transduction molecules such as c-SRC, c-RET, and oncogenic RET showed risks for malignant transformation and/or progression arose from arsenic and barium. Finally, our original hydrotalcite-like compound was proposed as a novel remediation system to effectively remove arsenic and barium from well drinking water. Hopefully, comprehensive studies consisting of (1) environmental monitoring, (2) health risk assessments, and (3) remediation will be expanded in the field of environmental health to prevent various disorders caused by environmental factors including toxic elements in drinking water.

## Background

Global heating has increased the regional differences in rainfall, resulting in increased water-deficient areas. Well drinking water is available in both rainy and dry seasons. Therefore, well water has been spotlighted as a stable source of drinking water. Since there are less pathogenic microbes in well water than in lake or pond water, the use of well water as drinking water has been accelerating in developing countries. Unfortunately, however, many kinds of disorders caused by toxic elements in well drinking water (element-originating diseases) have been reported in developing countries [1–5]. It is our urgent task to resolve the global issues that are directly associated with human life.

As the first step, environmental monitoring for well drinking water is essential to identify toxic elements contaminating in well water. In fact, elevated concentrations of toxic elements including arsenic, barium, manganese, iron, and uranium in well water were identified by our environmental monitoring in developing countries in Asia [6–13]. After considering the health risks for known toxic elements according to the health-based guideline levels for drinking water developed by WHO (World Health Organization), we could issue a global alert. However, element-originating diseases are supposed to be caused by exposure to unknown toxic elements that have no guideline values. Moreover, identification of novel element-originating diseases is not easy since most of the diseases are chronically developed and progressed [3, 8, 11]. Thus, an alert for known toxic elements in drinking water is insufficient to prevent the development of element-originating diseases.

As the second step, human studies including epidemiological [14–16] and clinical [17–21] studies are generally indispensable for assessing the risks of environmental factors including toxic elements for various diseases. Partial clarification of the mechanisms underlying the development of diseases is possible by analyses of human samples and physiological examinations in addition to questionnaires [2, 4, 5, 14–22]. However, there is an ethical limitation for detailed analysis of the mechanisms underlying the development of element-originating diseases in epidemiological and clinical studies. On the other hand, experimental studies using animals (in vivo) and cultured cells (in vitro) could be effective approaches for evaluating the pathogenic risks for environmental factors-originating diseases with elucidation of their mechanisms. Model animals for diseases might be useful for estimating the lesions caused by environmental factors and developing the preventive methods [23–33]. Cell physiological and biochemical examinations are also useful for health risk assessment in vitro [27–30, 34–41]. Expression and activity levels of proto-oncogene and oncogene products (protein tyrosine kinases) such as c-SRC, c-RET, and oncogenic RET have been shown to be correlated with malignant transformation from nontumorigenic cells to tumorigenic cells and with progression to further acquirement of malignant characteristics in transformed cells (carcinoma cells) [6, 37–47]. Therefore, expression and activity levels of molecules could be important clues to evaluate malignant transformation and progression in in vitro oncogenic risk analysis. Expression and activity levels of intracellular signal transduction molecules potentially sited downstream of protein-tyrosine kinases such ask MEK/ERK and PI3K/AKT can also be used for evaluation of oncogenic toxicities in in vitro studies [34–36]. Since protein tyrosine kinases also affect the biology of skin and neurons, their expression and activity levels are useful for estimating the risk of disturbance of sensory organs such as skin pigmentation and hearing [23, 24, 26]. Thus, more solid health risk assessment for element-originating diseases could be realized by the combination of in vivo and in vitro experimental studies and epidemiological and clinical studies for humans. However, environmental monitoring and health risk assessments are not sufficient for resolving the global issue of element-originating diseases.

As the third step, the development of suitable remediation systems for toxic elements after environmental monitoring and health risk assessments is essential for preventing element-originating diseases. Removal of trivalent arsenic in well drinking water has been difficult. The development of cheap and effective remediation systems that can be used for removal of trivalent arsenic and pentavalent arsenic and other toxic elements is required [9, 48].

**Fig. 1:**
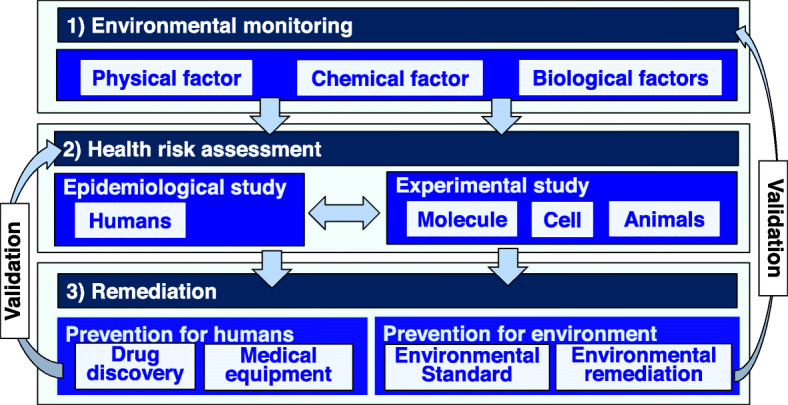
Comprehensive studies on environmental monitoring, health risk assessments, and remediation. The processes of comprehensive studies in environmental health and preventive medicine are presented. Environmental monitoring is indispensable as the first step for accurate analysis of environmental pollution. Experimental studies and epidemiological and clinical studies are the need for accurate assessments of the health risks of environmental factors. Finally, remediation should be performed to prevent environmental factor-mediated diseases

Specialization and segmentation of studies in the field of environmental health and preventive medicine seem to be proceeding. Comprehensive studies on (1) environmental monitoring, (2) health risk assessments, and (3) remediation may not be a main stream in the field. In this review article, results of studies using multidisciplinary approaches (Fig. 1) on oncogenic toxicities and disturbances of sensory organs (skin and ear) induced by arsenic and barium are introduced.

## Results

### Comprehensive studies on arsenic

#### Environmental monitoring for arsenic pollution of drinking well water

Fieldwork studies focusing on well drinking water have been performed in Asian countries including Bangladesh [8–11], Vietnam [6, 12], Malaysia [7], and Afghanistan [13]. Those studies showed that arsenic levels exceeded the WHO health-based guideline level for drinking water in these countries, suggesting that well drinking water is polluted with arsenic in large areas of Asian countries.

#### Assessments of the health risks of arsenic for skin hyperpigmentation and cancer

Various kinds of skin lesions including hyperpigmentation and cancer develop in people drinking arsenic-polluted water. Although information is increasing, information on skin diseases caused by exposure to arsenic [49–54] remains insufficient.

**Fig. 2:**
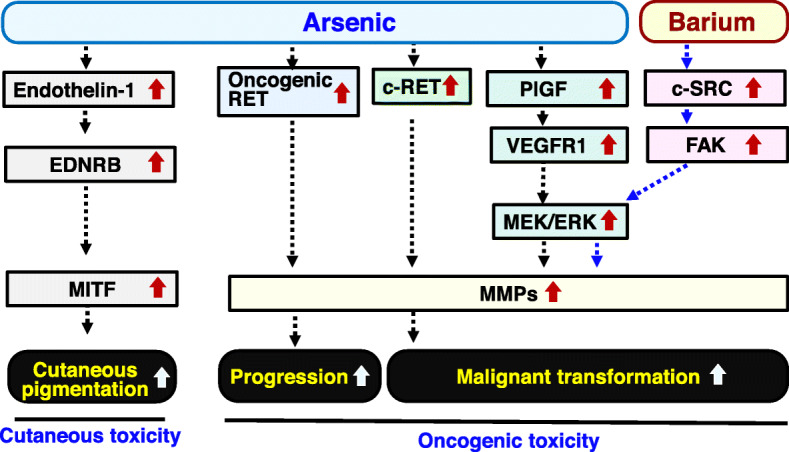
Biochemical analyses of cutaneous and oncogenic toxicities of arsenic and barium. Arsenic and barium may promote the development of hyperpigmented skin and malignant transformation and progression via activation of various intracellular signal transduction molecules. EDNRB endothelin receptor B, MITF microphthalmia-associated transcription factor, PlGF placental growth factor, VEGFR1 vascular endothelial growth factor receptor1, MEK mitogen-activated protein kinase, ERK extracellular signal–regulated kinase, MMPs matrix metalloproteinase, FAK focal adhesion kinase

Hyperpigmented skin is a hallmark for symptoms in patients with arsenicosis. However, the mechanism of arsenic-mediated hyperpigmented skin remains unclear. In our previous in vivo study, development of histologically detected hyperpigmented skin was observed in hairless mice that drank water containing 3 and 30 μM arsenic for 2 months [55]. A more than 5-fold increase in endothelin-1 (ET-1) expression level via NF-kappa B activation in the epidermis was found in the mice. Results of our in vitro study further confirmed that interaction between ET-1 in keratinocytes and endothelin receptor B (EDNRB) in melanocytes is correlated with arsenic-mediated development of skin pigmentation via microphthalmia-associated transcription factor (MITF) (Fig. 2) [55]. The effects of arsenic levels in cutaneous appendicular organs (hair and toenails) on skin pigmentation levels in the forehead (a sunlight-exposed area) and soles of the feet (sunlight-unexposed areas), which were digitally evaluated by using a reflectance spectrophotometer (L*-value), were investigated in our epidemiological study conducted in 150 Bangladeshi people. Significant correlations of the duration of drinking well water with arsenic levels in hair (*r* = 0.63, *p* < 0.01) and toenails (*r* = 0.60, *p* < 0.01) indicated that there is an accumulation of arsenic caused by drinking arsenic-polluted water [3, 9]. Our multivariate analysis showed that the duration of drinking well water and arsenic levels in the hair and toenails were significantly correlated with L* values of the forehead but not those of the sole. Thus, our study demonstrated that increased arsenic level promotes digitally evaluated hyperpigmentation of the forehead skin in humans [3].

Cancer is one of the most important diseases caused by drinking arsenic-polluted water. Our in vitro biochemical studies showed that arsenic promoted activities of oncogenic RET products as well as c-RET proto-oncogene products (RET-MEN2A protein and RET-PTC1 protein) via conformational modification of RET protein [37, 38]. The cysteine targeted by arsenic-mediated conformational modification of RET protein was also proposed [37, 38]. Arsenic increased secretion of matrix metalloproteinases (MMPs) in not only c-RET proto-oncogene-introduced cells but also RET oncogene-introduced cells [38]. Results of our biochemical studies suggest that arsenic is involved in both malignant transformation and progression [37, 38] (Fig. 2). Our in vitro experimental studies further showed that arsenic increased transforming activity with increases in the expression and secretion of placental growth factor (PlGF), a ligand of vascular endothelial growth factor receptor1 (VEGFR1), and an increase in VEGFR1/mitogen-activated protein kinase/ERK kinase (MEK)/extracellular signal-regulated kinase (ERK) activities in human nontumorigenic HaCaT skin keratinocytes. The arsenic-mediated increase in transforming activity was suppressed by decreased PlGF expression with decreased activities of the PlGF/VEGFR1/MEK/ERK pathway. Thus, PlGF might play a role in arsenic-mediated skin cancer development (Fig. 2) [1]. We therefore performed a fieldwork study focusing on urinary PlGF levels in Bangladeshi residents of cancer-prone areas. As expected, the urinary PlGF levels in residents of cancer-prone areas with high levels of arsenic in well drinking water were significantly higher than those in residents of a control area with limited pollution of arsenic in well drinking water [1].

Our results of both experimental and epidemiological studies suggest that urinary PlGF level is a potential biomarker for predicting arsenic-mediated development of skin cancer, though further research is needed.

#### Assessments of the health risks of arsenic for hearing

There is limited information on the correlation between hearing level and oral exposure to arsenic in both mice and humans. The hearing level of young mice treated with arsenic (22.5 mg/L) through drinking water was examined in our experimental study. It was found that hearing loss occurred with accumulation of arsenic in the inner ears of the mice treated with arsenic. A decreased number of auditory neurons and fibers were found in the organ of Corti from mice that had been exposed to arsenic ex vivo [5]. The correlation between hearing level and exposure to arsenic was examined in residents of Bangladesh who were aged 12–29 years. Multivariate analysis in our epidemiological study showed that hearing levels at high frequencies (4 k Hz, 8 k Hz, and 12 k Hz) in residents who were drinking arsenic-polluted water were significantly exacerbated compared to those in control residents. Taken together, the results of our epidemiological study and experimental study suggest that hearing level was decreased by drinking water polluted with arsenic in young people as well as in young mice [5].

Our further in vivo experimental study indicated a significant correlation between arsenic levels in inner ears and nails (*r* = 0.8113, *p* = 0.0014) in mice that had been orally exposed to arsenic. Multivariate analysis in our epidemiological study indicated that arsenic levels in toenails, but not those in urine, were significantly correlated with hearing levels at high frequencies (4 k Hz, 8 k Hz, and 12 k Hz) in 145 subjects aged 12–55 years in Bangladesh. Since our results in mice suggest that nail arsenic level could be an index for the arsenic level in inner ears, the level of arsenic in nails is a potential biomarker for hearing loss caused by an accumulation of arsenic in inner ears in humans [56].

Thus, our combined results of experimental and epidemiological studies suggest that hearing is impaired by exposure to arsenic in drinking water in humans.

#### *Remediation for arsenic in well drinking water*

Not only the WHO health-based guideline value (10 μg/L) for arsenic in drinking water but also our results for carcinogenic, cutaneous, and neural toxicities of arsenic suggest that there is an urgent need for the development of a remediation system for arsenic in well drinking water. In our previous study, we developed a hydrotalcite-like compound as an adsorbent for trivalent arsenic as well as hexavalent arsenic [48]. We found that the adsorbent could reduce levels of iron and uranium as well as the level of arsenic in well drinking water in Asian countries [13, 48].

### Comprehensive studies for barium

#### Environmental monitoring for barium pollution of drinking well water

An increased incidence of cancer has been reported in more than 36 million patients with arsenicosis caused by drinking arsenic-polluted well water [3, 9]. High levels of barium as well as arsenic have been found in well water in previous studies [9, 57]. However, there is limited information about the carcinogenic toxicity of barium intake from drinking water.

#### Assessments of the risks of barium for carcinogenesis

There is very limited information about the carcinogenic toxicity of barium. In our in vitro experimental study, we investigated the effects of exposure of barium for a short period (≤ 4 days) on human nontumorigenic HaCaT keratinocytes [58]. Our study showed that barium (5–50 μM) promoted anchorage-independent growth and invasion of HaCaT keratinocytes. Barium at a concentration of 5 μM increased that activities of regulators for anchorage-independent growth and/or invasion (c-SRC, FAK, ERK, and MT1-MMP) in nontumorigenic cells (HaCaT, NIH3T3, and melan-a cells) but not in tumorigenic cells (HSC5 and A431 cells). These results suggest that barium promotes the transforming activity of various nontumorigenic cells (Fig. 2) [6]. In our further in vitro experimental study, we investigated the effect of exposure to 5 μM of barium for a long period (4 months and 37 passages) on invasion ability of HaCaT keratinocytes. Activities of cell invasion and focal adhesion kinase (FAK) and expression level of matrix metalloproteinase (MMP) in HaCaT keratinocytes after treatment with barium for a long period were significantly higher than those of untreated keratinocytes. Since invasion ability is one of the malignant characteristics, exposure to a low level of barium for a long time may increase the malignant characteristics of nontumorigenic keratinocytes [59]. Thus, our in vitro studies suggest a potential carcinogenic toxicity of barium (Fig. 2).

#### *Remediation for barium*

Considering the WHO health-based guideline value (700 μg/L) for barium in drinking water and the potential carcinogenic toxicity of barium, a remediation system to remove barium from drinking water is required. Our original adsorbent of a hydrotalcite-like compound could decrease the level of barium in well water in Asian countries to less than 7 μg/L in a short time (< 1 min) [9]. Thus, we newly demonstrated a cheap and high-efficacy remediation system that is useful for removal of toxic elements in well drinking water. We also demonstrated that melanin could bind barium and other elements in mice, cultured cells, and cell-free systems [27, 28]. Since synthesized melanin is expensive, its practical realization may be difficult as a novel remediation system for elements in drinking water.

## Discussion

At present, there is limited in vivo evidence for oncogenic toxicities of arsenic and barium in our study of health risk assessment, though animal studies are useful for oncogenic risk assessment in vivo [60–63]. Previously, we developed *RET*-transgenic mice (RET-mice) carrying oncogenic *RET* in which hyperpigmented skin and skin benign and malignant tumors developed [64–68]. The influences of environmental factors on the process of malignant transformation and progression could be investigated in the RET-mice because their dynamics could be macroscopically observed in the mice [69–73]. Therefore, we need to investigate in vivo influences of arsenic and barium on malignant transformation and progression in the RET-mice in the future. In addition to animal experiments, epidemiological studies are needed to evaluate the influences of barium on malignant transformation in humans.

More importantly, practical realization and dissemination of suitable remediation systems including our hydrotalcite-like compound should be promoted in developing countries that suffer from elements-originating diseases. In the process, the cooperation of health services in developing countries and the cooperation of companies that can produce large amounts of the adsorbent will be required.

## Conclusion

In this review article, our comprehensive studies on (1) environmental monitoring, (2) health risk assessments, and (3) remediation in environmental health and preventive medicine are presented (Fig. 1). Comprehensive studies might provide various new insights that cannot be obtained by specialized studies. Therefore, we hope that comprehensive studies will be more general in the field of environmental health and preventive medicine.

## Data Availability

All data and materials in this paper are available from the corresponding author on reasonable request.

## Abbreviations

EDNRB: Endothelin receptor B
ERK: Extracellular signal–regulated kinase
ET-1: Endothelin-1
FAK: Focal adhesion kinase
MEK: Mitogen-activated protein kinase/ERK kinase
MITF: Microphthalmia-associated transcription factor
MMP: Matrix metalloproteinase
PlGF: Placental growth factor
RET-mice: RET-transgenic mice
VEGFR1: Vascular endothelial growth factor receptor1
WHO: World Health Organization
